# Ground Testing Strategies for Verifying the Slew Rate Tolerance of Star Trackers

**DOI:** 10.3390/s140303939

**Published:** 2014-02-26

**Authors:** Tom Dzamba, John Enright

**Affiliations:** Department of Aerospace Engineering, Ryerson University, 350 Victoria Street, Toronto M5B 2K3, Canada; E-Mail: jenright@ryerson.ca

**Keywords:** star trackers, slew tolerance, attitude availability, star detection, attitude determination

## Abstract

The performance of a star tracker is largely based on the availability of its attitude solution. Several methods exist to assess star tracker availability under both static and dynamic imaging conditions. However, these methods typically make various idealizations that can limit the accuracy of these results. This study aims to increase the fidelity of star tracker availability modeling by accounting for the effects of detection logic and pixel saturation on star detection. We achieve this by developing an analytical model for the focal plane intensity distribution of a star in the presence of sensor slew. Using the developed model, we examine the effects of slew rate on star detection using simulations and lab tests. The developed approach allows us to determine the maximum slew rate for which a star of a given stellar magnitude can still be detected. This information can then be used to describe the availability of a star tracker attitude solution as a function of slew rate, both spatially, across the entire celestial sphere, or locally, along a specified orientation track.

## 1. Introduction

Satellites that require high accuracy attitude estimates (<1 arc-min) generally employ the use of star trackers. These sensors operate by taking images of the star field and matching observed patterns to an onboard catalog. For most star trackers, the availability of this attitude measurement is generally greater than 99% in ideal conditions [1]. However, in many cases, satellites are required to change their attitude, either continuously, as with Earth observation (EO) satellites, or periodically, as with space telescopes. For star trackers onboard such satellites, angular motion during imaging (slew) causes stars to smear out over a larger number of pixels than they would occupy in static imaging conditions. This reduces the signal-to-noise ratio (SNR) of imaged stars, which decreases the detection performance of dim stars. Detecting less stars in each image ultimately impairs the accuracy and the availability of a star tracker attitude solution. Each star tracker claims to be tolerant of some amount of sensor slew; however, it is challenging to quantify the exact impact this angular motion has on sensor performance.

This paper investigates the effects of slew rate on the availability performance of a star tracker. Specifically, we develop an analytical model of the intensity distribution of a star smear. We combine this model with star detection logic in a simulation-based approach to evaluate the effects of slew rate on star tracker availability. We verify these results through lab testing and discuss further verification using field tests. Lastly, we propose two new measures of star tracker availability that both incorporate the effects of slew rate and represent improved modeling fidelity. Although the numerical results of this paper are specific to the Sinclair Interplanetary ST-16 star tracker, the models and methods developed are applicable to any star tracker with only minor modifications.

Before we can begin discussing slew rate tolerance, we need to understand how sensor slew impacts the performance of a star tracker. The remainder of this section defines star tracker availability, introduces our test sensor and outlines the methods we use to measure detection performance as a function of slew rate.

### 1.1. Star Tracker Availability

The performance of a star tracker is generally described by two parameters: availability and accuracy. Accuracy is defined as the uncertainty in the attitude estimate. Availability is defined as the fraction of the celestial sphere, also known as firmament, over which a reliable attitude solution is possible. In this study, we only examine the effects of sensor slew on availability. For more information on how sensor slew affects star tracker accuracy, please see [2–6].

The key requirement for a star tracker attitude solution is detecting a sufficient amount of stars in each image to form an unambiguous star pattern required for matching. The required number of detected stars, which we denote as *N_min_*, varies depending on the operating mode of the star tracker and the performance of the matching algorithm. If no previous attitude information is known, at least three stars are required to solve the lost-in-space (LIS) problem using star tracker measurements. This limit of three stars stems not from the solution for attitude using vector observations, which only requires two stars [7,8], but from the identification of stars within an image [9]. If only two stars are detected in an image, typically not enough information is known to identify one star from another. Therefore, at least one additional star is required.

This lower bound of *N_min_* = 3 represents the most optimistic case, which implies the matching algorithm can correctly identify each star based on the respective three-star pattern. Due to pattern ambiguity in the star catalog, this lower bound is commonly increased to *N_min_* = 4, which is a more conservative representation of matching performance. Once the attitude of the spacecraft is known, the star tracker can switch into a tracking mode. In this mode, only two stars are generally required in each image to determine the incremental change in attitude between sequential images (*N_min_* = 2). For this study, we assume that pattern ambiguity is not a limiting factor and define the availability of an attitude solution by *N_min_* = 3. One problem with this definition is that it conflates stochastic effects (star detection) with non-stochastic effects (star distribution, slew rates, tracking modes, etc.) and, therefore, is difficult to quantify over a range of operating conditions.

**Figure 1.**
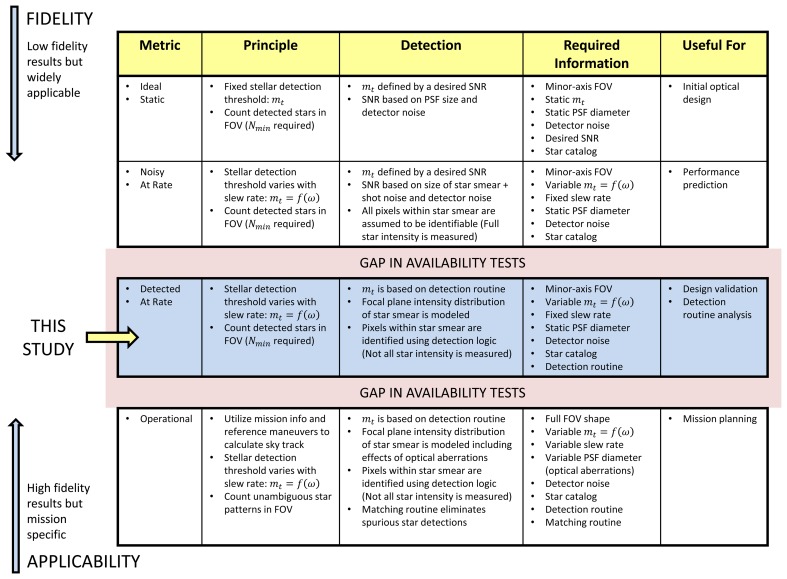
Commonly used types of availability testing.

Throughout the design and development process of a star tracker, several different models are used to predict the availability performance of the sensor. The lowest fidelity models generally assume idealized (static) imaging conditions and are useful for examining the top level performance of candidate optical systems [1,4]. These models are typically based on a fixed stellar detection threshold, *m_t_*, which is used in conjunction with the sensor field of view (FOV) to determine the number of detectable stars for a given sensor orientation. Repeating this calculation over a large number of orientations, equally spaced across the celestial sphere, yields an idealized measure of star tracker availability. The fixed *m_t_* is typically defined by a minimum SNR set by the noise of the image detector and the size of the sensor's point spread function (PSF). This type of model is summarized by the first row of Figure 1.

A step up from the lowest fidelity are various models that explicitly include the effects of slew rate. These models utilize a dynamic stellar detection threshold that is based on the slew rate, *m_t_* = *f* (*ω*), and a minimum star SNR [10,11]. These models account for the size of the smear, but do not explicitly consider the intensity distribution within the smear itself. The typical assumption with these models is that all of the starlight incident on the image detector is detected. In reality, the measured intensity is less than the modeled star intensity, due to the effects of pixel saturation and star detection logic. Pixel saturation has the effect of masking image intensity, due to the bit depth of the analog-to-digital converters (ADCs) of the image detector. Star detection logic is used to detect candidate stars and separate the star image from the background image noise. Similar to the model described in the first row of Figure 1, the detection of a specific star is still defined by a minimum SNR. However, in this case, the SNR is based not only on the noise of the image detector and the size of the PSF, but on the length of the star smear. These models are summarized by the second row of Figure 1.

On the opposite end of the fidelity spectrum, we have various high fidelity models. These models produce more accurate results, but they rely on specific information about mission orbits and maneuvers. Availability is measured along the specific orientation track the sensor will follow on the celestial sphere. This track is defined by the dynamics of the spacecraft. Star detection is assessed by the exact detection routines employed on the star tracker. These models can include the effects of optical aberrations on the PSF, as well as the effects of bright bodies (Sun, Moon, other planets). Furthermore, these models would typically revise the definition of availability from having at least *N_min_* detectable stars in the FOV to having a detectable non-ambiguous star pattern in the FOV, which contains enough stars for star identification. These models are summarized by the last row of Figure 1 and would typically be used to predict the availability performance of a spacecraft following launch.

There is currently a gap in available performance models between those which yield high fidelity results and those which are not specific to a particular mission. This work attempts to bridge this gap and provide some intermediate models of availability. The aim is to increase the fidelity of the availability model while not limiting its applicability to any specific mission. We explicitly consider the effects of sensor slew on the focal plane intensity distribution of a star. This allows us to incorporate the effects of pixel saturation and star detection logic on the measured intensity of a star, increasing the accuracy of predicted star SNR. We also examine the commonly modeled effects of star distribution on star tracker availability. Figure 1 summarizes the metrics, required knowledge and potential application of the common types of availability testing.

For the purpose of this paper, we define three types of availability that we use to describe the transition from the general and heavily idealized, to the mission-specific models of availability shown in Figure 1:

1. Spatial Static availability. There is no motion of the sensor during an image exposure. The availability is calculated using a large set of discrete sensor orientations that are equally spaced along the celestial sphere. At each orientation, we determine if at least *N_min_* = 3 stars are detected based on the sensor FOV and a fixed stellar detection threshold, *m_t_*. This definition represents the idealized static model described by the first row of Figure 1.
2. Spatial Dynamic availability. The sensor is moving at a constant rate during image exposure. Availability is still evaluated at discrete sensor orientations; equally spaced along the celestial sphere, but now, with a detection threshold dependent on the slew rate, *m_t_* (*ω*). Unlike the second row of Figure 1, detection is not based on the ideal SNR, but the actual SNR, as measured by the image detector and the detection logic employed by the star tracker. This represents the model described by the third row of Figure 1.
3. Along-track Dynamic availability. The sensor is moving at a constant rate during image exposure. Availability is calculated only along the specific path (orientation track) and at specific slew rates the sensor orientation will follow as a result of mission dynamics. Similar to spatial dynamics availability, detection is determined by detection logic employed by the star tracker. This represents an approximate version of the bottom row of Figure 1.

### 1.2. The ST-16 Star Tracker

**Figure 2.**
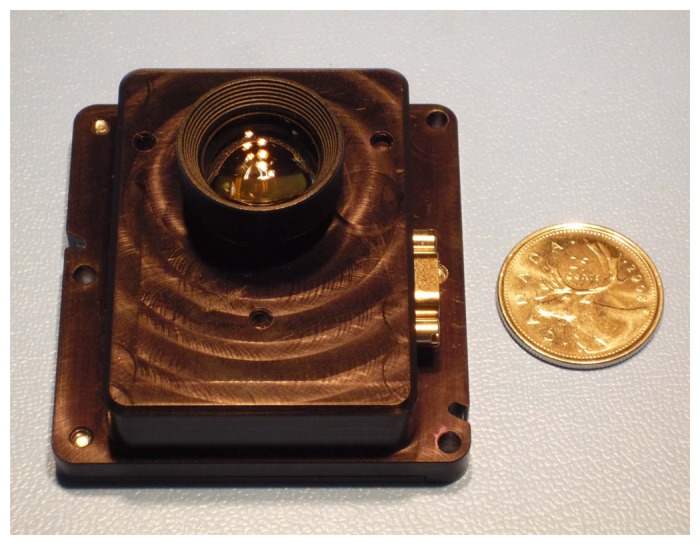
The ST-16 star tracker with a Canadian quarter (25(image)).

For this study, we have used the Sinclair Interplanetary ST-16 star tracker as our baseline sensor. The ST-16 is a relatively new nanosatellite-class star tracker that became available in 2011. An image of the unit is shown in Figure 2 and some key specifications are listed in Table 1. All of our test results reflect some preliminary performance characteristics of this device, but our approach to verifying slew rate tolerance is generalizable to other star trackers. For more information on the ST-16, please see Enright *et al.* [12] or Dzamba *et al.* [13].

**Figure 3.**
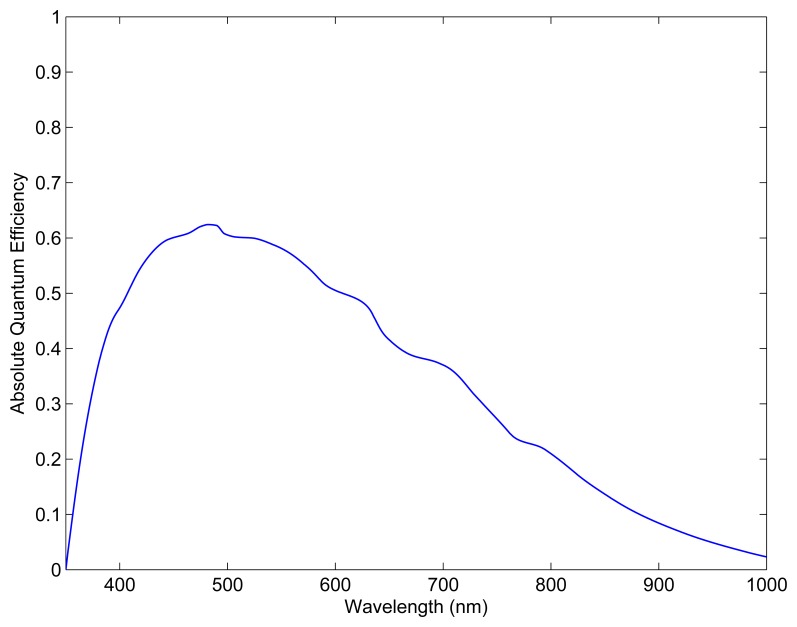
Aptina MT9P031 quantum efficiency.

The image detector used onboard the ST-16 star tracker is the Aptina MT9P031 complementary metal-oxide-semiconductor (CMOS) detector. The quantum efficiency of the MT9P031 is shown in Figure 3 [14]. The ST-16 star catalog contains all stars of a visual magnitude of 5.75 or brighter (3, 746 stars in total), drawn from the Yale Bright Star catalog (YBS) [15]. Using *m_t_* = 5.75 as the stellar detection threshold in static conditions, in combination with the ST-16 half-axis FOV of 7.5°, we can calculate the spatial static availability of the ST-16 attitude solution to be > 99.9%. This is calculated by testing a large number of sensor orientations (10,000) for at least *N_min_* = 3 detectable stars in the FOV. The tested orientations are distributed evenly across the celestial sphere using the method described by Marantis [16].

**Figure 4.**
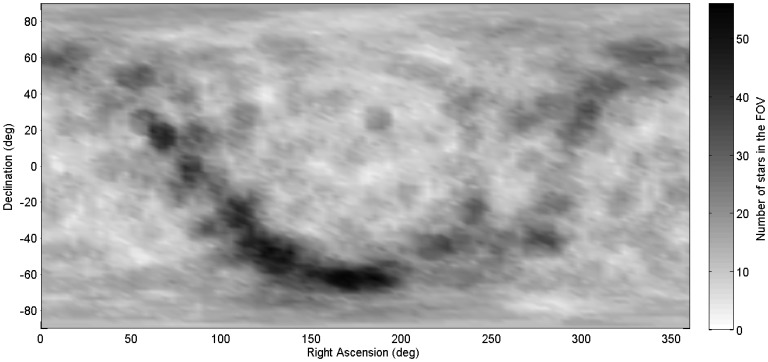
ST-16 spatial static star distribution map (0 deg/s).

**Figure 5.**
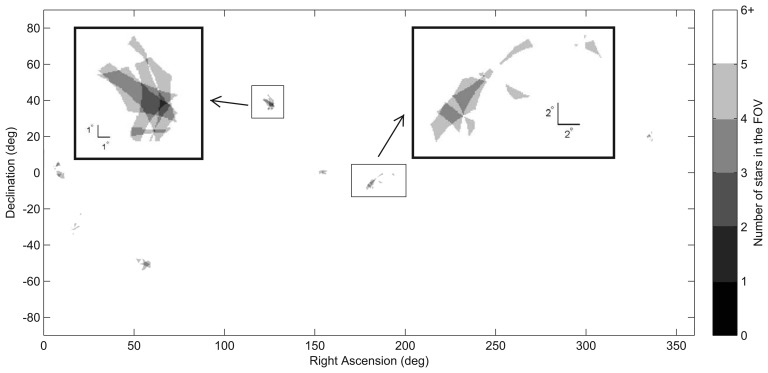
Sky regions with poor ST-16 star availability (0 deg/s). Note: the color axis is flipped with respect to Figure 4 to increase the visibility of star-sparse regions.

Figures 4 and 5 show a distribution map of the number of stars within the ST-16 FOV as a function of sensor orientation. From this figure, we can see how uneven the star distribution is across the celestial sphere. When pointing near the galactic equator, more than 10 stars in the FOV are typical. Conversely, in the neighborhood of the galactic poles, many views see only three stars (see Figure 5). These regions of sparse star distribution are directly dependent on the sensor FOV and the range of detectable stellar magnitudes.

One of the main contributions of this work is that it enables the incorporation of detection logic into the availability analysis of a slewing star tracker. To examine the importance of this addition, this paper utilizes the detection scheme used onboard the ST-16 star tracker. This detection scheme can be summarized by describing three threshold values:

- Lit pixel. This value defines the minimum intensity of an image pixel that is considered to be lit by starlight, as opposed to just sensor noise. Pixels that are above this threshold are labeled *lit pixels*. The lit pixel threshold used for this study was 120 detector counts out of a possible 4,095 (constrained by the ST-16 image detector's 12-bit ADCs).
- Number of contiguous pixels. This defines the minimum number of contiguous lit pixels that each candidate star must possess before it can be considered as a valid detection. For this study, we require at least six contiguous pixels.
- Integrated intensity. This value describes the minimum integrated intensity (summed intensity) of all contiguous lit pixels that compose a candidate star. Candidate stars above this threshold are considered valid detections. For this study, we define the integrated intensity threshold as 1,000 detector counts.

### 1.3. Testing the Performance of Star Detection

**Figure 6.**
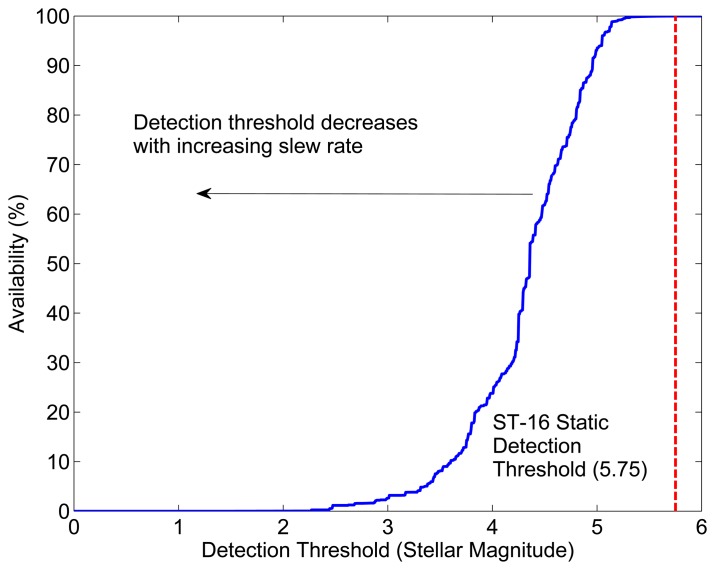
The ST-16 spatial static availability for varying stellar detection thresholds.

The sensor slew during an image exposure spreads the light from each star over a larger region of the detector than compared to static imaging conditions. Given that the areal density of the detector noise is approximately constant, as the incoming light spreads over more pixels, the integrated SNR over a star image drops. Typical star tracker image processing routines subtract out most of the detector dark response, so the SNR reduction appears as a decrease in the apparent brightness of imaged stars. At some point, the integrated intensity (summed detector response) of a star will drop below the threshold of reliable detection. Therefore, as the slew rate increases, the range of stellar magnitudes that the star tracker can detect decreases. This effectively reduces the number of stars in the working catalog, ultimately leading to a drop in availability. Figure 6 shows the change in availability of the ST-16's attitude solution for various limiting stellar detection thresholds.

As part of this paper, we examine the efficiency of three methods that we use to determine the performance of star detection as a function of slew rate. The results from these methods can be used to define the stellar magnitude threshold in terms of slew rate, *m_t_* = *f* (*ω*), which can then be related to the availability of the attitude solution. The methods we examine are:

- Simulations. We utilize analytical models to calculate the shape and intensity of an imaged star in the presence of sensor slew. This is combined with the detection scheme implemented onboard the ST-16 to determine the rate at which a star of a given magnitude would cease to be detected.
- Lab Tests. The ST-16 star tracker is placed onto a three-axis motorized gimbal capable of slewing at a prescribed rate. While slewing, images are taken of a star source. This strategy represents an improved level of realism, as the actual star tracker and onboard routines are used for image formation and processing.
- Field Tests. This strategy involves moving the star tracker at fixed rates while collecting nighttime sky images. Atmospheric factors introduce variability and attenuation into images taken with this strategy. These effects can be minimized with the selection of an observation site with good viewing conditions, a high-accuracy telescope drive system and the use of atmospheric extinction models.

Each of these methods represents a different level of realism for a different cost (material and labor). We compare the performance of these methods and examine which method is the most beneficial at different points of the sensor development process.

## 2. Modeling the Effects of Sensor Slew on Star Tracker Imaging

Since stars are effectively point sources of light, the shape of an imaged star is commonly approximated by the point spread function (PSF) of the sensor's optical system. During static imaging conditions, this is typically modeled by a symmetric two-dimensional Gaussian distribution. In the presence of sensor slew, the symmetric 2D Gaussian is elongated along the direction of motion, forming a blurred image, which we denote as the *star streak*.

This section begins by discussing the effects of slewing in different directions on star detection. Following this, we review two analytical models from the literature that describe the intensity of a star. We develop a new analytical model for the shape of a star image, taken at a given rate. We then combine these models with shot noise and detector read noise to simulate a realistic star image. Lastly, we apply detection logic to determine the tolerable slew rate for a given stellar magnitude.

### 2.1. Types of Sensor Slew

It is useful to separate the slew rate of a star tracker into two components: a roll component (*ω_z_*) and a cross-axis component (*ω_xy_*). Each component results in a different effect, with a different relative magnitude. If we neglect the effects of optical aberrations and consider a pinhole imaging model, a pure cross-axis rotation (*ω_z_* = 0) results in linear star streaks on the focal plane of the star tracker. The length of each streak, Δb, is dependent only on the magnitude of the slew rate, $\left\|\omega_{xy}\right\|=\sqrt{\omega_{x}^{2}+\omega_{y}^{2}}$, the exposure time, *t_e_*, and the focal length of the sensor optics, *f*. The length of a star streak for a pure cross-axis slew is given by:

(1) $$\Delta {\text{b}}_{\text{ca}}=f\tan(\left\|\omega_{xy}\right\|t_{e})$$

For a pure boresight roll, stars will appear as streaks in the shape of circular arcs, centered about the axis of rotation. Similar to the cross-axis case, the length of each arc is dependent on the magnitude of the roll rate, ‖*ω_z_*‖, and the exposure time. However, instead of scaling directly with focal length, the arc length scales with distance from the axis of rotation (in this specific case, the boresight). Practically, the largest distance from the boresight that a star can be detected at rate is at the edge of the minor-axis FOV. If we let *h* denote the minor dimension of the image detector, the maximum arc length of a star streak is calculated using:

(2) $$\Delta {\text{b}}_{\text{r}}=(h/2)\tan(\left\|\omega_{z}\right\|t_{e})$$

Since the focal length of the optics is always significantly larger than the dimension of the detector, we can see that cross-axis slew rates will cause larger streak lengths. Comparing Equations (1) and (2), we can see that even at the largest off-axis distance, *h*/2, the cross-axis streak length is still larger than the roll streak length by a factor of *2f*/*h*. For the ST-16, this factor works out to be ≈ 7.5.

Therefore, a pure cross-axis slew produces the maximum streak length for a given slew rate. Since streak length is the primary factor that impacts star detection, for the remainder of this paper, we limit our investigation of slew rate on star detection to pure cross-axis slews only.

### 2.2. Intensity Model

Various sensitivity models exist in the literature for predicting the number of photoelectrons observed by an image detector for a star of a given magnitude. All of these models predict identical trends in detected star intensity as a function of stellar magnitude. However, they typically differ by a scaling factor due to various assumptions (spectral class, passband, *etc.*) made when determining the photon flux from a zero-magnitude reference star. Since the slew tolerance for a star of a given magnitude is directly dependent on the observed photon flux from that star, the intensity model chosen will affect the calculated tolerable slew rate. However, the aim of this paper is to improve the fidelity of slew tolerance modeling by better accounting for *changes* in observed star intensity as a function of slew rate, as opposed to predicting exact performance values in static conditions. Therefore, we adopt two different sensitivity models (one conservative and one liberal) from the literature and use them to establish bounds on slew tolerance performance.

The first sensitivity model we use is the model presented by Liebe [1]. We provide a brief review of the model below. Please see [1] for more information. The spectral radiance from a black body, at a given wavelength and temperature, is given by:

(3) $$I(\lambda ,T)=\frac{\left(2\pi hc^{2}\right)}{\lambda^{5}(e^{hc/\lambda k_{b}T}-1)}$$

where *h* is Planck's constant = 6.626 × 10 ^−34^ J · s, *c* is the speed of light = 2.997 × 10^8^ *m/s, k_B_* is Boltzmann's constant = 1.38 × 10^−23^ J/K, λ is the wavelength and T is the temperature (in Kelvin). Equation (3) is then used to predict the spectral irradiance of a zero-magnitude reference star, *I_o_* (*λ*), with a surface temperature of 5, 800K. This is achieved by scaling the spectral radiance, *I* (*λ*), given by Equation (3), such that the total radiance (spectral radiance across all wavelengths) is 2.96 × 10^−14^ W/m^2^. This scale factor stems from the solar constant, 1.3 kW/m^2^, divided by 4.2 × 10^10^, to account for the discrepancy in brightness between the Sun and a star of *m* = 0. This spectral irradiance is then expressed as a photon flux by dividing *I_o_* (*λ*) by the photon energy *E* = *hc*/*λ*. This is given by:

(4) $$P_{o}(\lambda )=\frac{I_{o}(\lambda )}{hc/\lambda }$$

The fraction of incident photons that are converted into photoelectrons by the image detector is given by the absolute quantum efficiency, *n_Q_* (*λ*), of the image detector. The absolute quantum efficiency for the Aptina MT9P031 image detector is shown in Figure 3. Using *n_Q_* (*λ*) and the passband of the image detector (typically 400–800 nm), the number of detected photoelectrons, per unit area, per unit time, for a zero-magnitude reference star, can be calculated by:

(5) $$S_{o}=\int_{\lambda_{1}}^{\lambda_{2}}n_{Q}\left(\lambda \right)P_{o}(\lambda )\text{d}\lambda$$

where *λ*_2_ and *λ*_1_ represent the upper and lower extents of the image detector passband, respectively.

Using the result of Equation (5), which has the units of photoelectrons/ (*s* · m^2^), we can now calculate the number of detected photoelectrons for a star of a desired magnitude using the star tracker aperture and the exposure time.

(6) $$S_{e}^{\text{Liebe}}=\frac{1}{4}t_{e}\pi D^{2}\frac{S_{o}}{2.5^{m}}$$

where *D* is the diameter of the star tracker aperture, *t_e_* is the exposure time and *m* is the apparent magnitude of the star.

The second sensitivity model we use is the model presented by Enright *et al.* in [17]. This model is based on a constant calculated by Reed [18] that states the photon flux from a type A, zero-magnitude reference star is *ϕ*_0_ = 1.8 × 10^10^ photons · *m*^−2^ · s^−1^. Using this, we can determine the number of photo-electrons that would be detected by the ST-16 star tracker for any star of known magnitude using:

(7) $$S_{e}=\eta_{Q}t_{e}A\phi_{0}\cdot 10^{\frac{-2}{5}(m_{1}-m_{o})}$$

where *η_Q_* is the mean quantum efficiency of the detector across the selected passband (400–800 nm), *t_e_* is the observation time, *A* the aperture area, *ϕ*_0_ the stellar flux from a reference star and *m*_1_ and *m*_0_ are the apparent stellar magnitudes of both the star in question and the reference star. Expressing *A* in terms of the aperture diameter, *D*, gives:

(8) $$S_{e}^{\text{Reed}}=\frac{1}{4}\eta_{Q}t_{e}\pi D^{2}\phi_{0}\cdot 10^{-\frac{2}{5}m}$$

where *m*_1_ is now simply *m* and represents the stellar magnitude of a given star. The detector response, in terms of digital counts, can be determined from the number of detected photoelectrons by multiplying *S_e_* by a factor of 6.5. This factor is based on the operating gain set to 16, and the ADCs of the MT9P031 detector [19].

**Figure 7.**
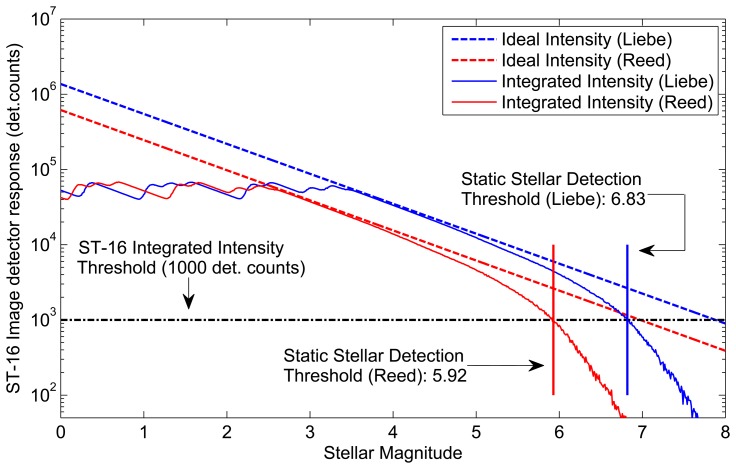
Comparing star tracker sensitivity models.

Figure 7 compares the ideal intensity from each model and the corresponding integrated intensities (intensity as detected by ST-16). Comparing the ideal intensity to the integrated intensity, we see similar trends for either model. There is a specific stellar magnitude where the ideal intensity, and the ST-16 integrated intensity is almost identical. Then, on either side of this specific stellar magnitude, we see the integrated intensity decrease compared to the ideal intensity. Although the trends are similar, the mechanisms for this discrepancy are different for bright stars and dims stars. For bright stars, the discrepancy is caused by the effects of pixel saturation. For dim stars, the discrepancy is caused by the ST-16 detection logic, excluding more of the star image, as individual pixel intensities fall closer to the noise floor

The Liebe sensitivity model predicts integrated intensities approximately twice that of the Reed sensitivity model. Although this is a significant discrepancy, we can use these models to predict bounds on star tracker performance. If you have on-orbit data from your star tracker, or perhaps even high quality field data (from an astronomical observatory), then you can utilize the actual observed intensity from a reference star to tune these models.

### 2.3. Shape Model

The PSF of a star from a non-rotating spacecraft can be approximated as a symmetric Gaussian distribution. The intensity distribution on the focal plane can be modeled by the function, *S*(*x*, *y*), given as:

(9) $$\begin{array}{ll} S(x,y) & =\frac{S_{e}}{2\pi {\sigma_{s}}^{2}}e^{-\left[\frac{{\left(x-\mu_{x}\right)}^{2}+{\left(y-\mu_{y}\right)}^{2}}{2{\sigma_{s}}^{2}}\right]}\\ & =\frac{S_{e}}{2\pi \sigma_{s}^{2}}e^{-\frac{r^{2}}{2{\sigma_{s}}^{2}}} \end{array}$$

where *S_e_* is the modeled intensity of the imaged star given by Equations (6) or (8), *σ_s_* describes the size of the PSF (it can be measured during calibration), (*μ_x_*, *μ_y_*) is the location of the star's centroid and **r** is the radial distance of a point (*x*, *y*) from the centroid. Equation (9) is commonly used to model the intensity distribution of a star for static imaging conditions. When the star tracker is moving, the centroid of an imaged star moves during the course of an exposure, forming an elongated streak. If we define the star vectors at the beginning and end of an exposure as **b** and **b′**, we can model this motion as an infinitesimal rotation through an angle, *t_e_* ***ω***, as described by:

(10) $${\text{b}}^{\prime }=({\text{I}}_{3\times 3}-t_{e}\omega^{\times })\text{b}$$

where *ω*^×^ denotes the skew-symmetric matrix of the angular velocity vector, ***ω***, and *t_e_* is the star tracker exposure time. The vector difference can be written as:

(11) $$\delta \text{b}={\text{b}}^{\prime }-\text{b}=-t_{e}\left[\omega^{\times }\right]\text{b}$$

In the operating regime of small slew rates (<10 °/*s*), we assume that the loci of the centroids in the detector plane appear as linear segments with displacement, Δ**b**, specified in pixels. This is given as:

(12) $$\Delta \text{b}=\frac{ft_{e}}{\gamma b_{z}}\left[\begin{array}{c} \omega_{z}b_{y}-\omega_{y}b_{z}\\ \omega_{x}b_{z}-\omega_{z}b_{x} \end{array}\right]$$

where *γ* denotes the pixel size. Using Equation (12), we can define the integrated response at a point (*x*, *y*) of the PSF as a function of the initial centroid position, *μ*_o_, and the focal plane displacement, Δ**b**. We can rewrite Equation (9) to include the elongation of the PSF as:

(13) $$S(x,y,t)=\frac{S_{e}}{2\pi \sigma_{s}^{2}}e^{-\frac{r{\left(t\right)}^{2}}{2\sigma_{s}^{2}}}$$

where *t* is the time from the beginning of the PSF exposure, and *r*^2^ from Equation (9) is now:

(14) $$r{\left(t\right)}^{2}={\left(x_{o}+\frac{\Delta b_{x}t}{t_{e}}-x\right)}^{2}+{\left(y_{o}+\frac{\Delta b_{y}t}{t_{e}}-y\right)}^{2}$$

The quantities *μ*_o_ = (*x_o_*, *y_o_*) are the focal plane coordinates of the star centroid at the beginning of the exposure and (*x*, *y*) are the coordinates of an arbitrary point of interest. Substituting Equation (14) into Equation (13), expanding and collecting like terms, we get:

(15) $$S(x,y,t)=\frac{S_{e}}{2\pi \sigma_{s}^{2}}e^{z}$$

where:

(16) $$z=a_{2}t^{2}+a_{1}t+a_{o}$$

and:

(17) $$a_{2}=\frac{\left(\Delta b_{x}^{2}+\Delta b_{y}^{2}\right)}{2\sigma_{s}^{2}t_{e}^{2}}$$

(18) $$a_{1}=\frac{\Delta b_{x}(x-x_{o})+\Delta b_{y}(y-y_{o})}{\sigma_{s}^{2}t_{e}}$$

(19) $$a_{0}=\frac{-({x_{o}}^{2}+x^{2}+{y_{o}}^{2}+y^{2})+2(x_{o}x+y_{o}y)}{2\sigma_{s}^{2}}$$

We now integrate Equation (15) with respect to time and get the focal plane intensity distribution of an imaged star in the presence of the slew rate:

(20) $$S_{b}(x,y)=\int_{0}^{t_{e}}S(t)dt=A_{o}B_{o}e^{\frac{1}{4a_{2}}4a_{0}a_{2}-a_{1}^{2}}$$

where:

(21) $$A_{o}=\frac{S_{e}}{2\pi \sigma_{s}^{2}}$$

(22) $$B_{o}=\frac{\sqrt{\pi }}{2\sqrt{-a_{2}}}\left[\text{erf}\left(\frac{1}{2}\frac{a_{1}}{\sqrt{-a_{2}}}\right)-\text{erf}\left(\frac{1}{2}\frac{2a_{2}\Delta t_{e}+a_{1}}{\sqrt{-a_{2}}}\right)\right]$$

Given an angular rate and a static star intensity, this derivation gives the shape and focal plane intensity distribution of a star imaged during sensor slew.

## 3. Star Detection at Rate

Using the analytical models developed in the previous section, we use simulations to examine how the measurable intensity of a star is affected by the slew rate of the sensor. We compare the results of these simulations with the results from a series of lab tests that utilize a motorized gimbal, an ST-16 engineering model and a star source. Lastly, we examine the potential accuracy and benefit of acquiring field results at a rate.

### 3.1. Simulation Tests

Using the developed analytical models for the shape and intensity distribution of a star, we conduct simulation tests to accomplish two primary objectives. The first objective is to examine the decrease in the measurable intensity of a star (integrated intensity), as a function of slew rate. The second objective is to determine the maximum slew rate at which a star of a given stellar magnitude can be detected. We denote this maximum slew rate as the tolerable slew rate for a given stellar magnitude. Each simulation consists of iteratively simulating a star image for a range of slew rates, adding some typical imaging noise sources and then applying the detection scheme equivalent to the one used onboard the ST-16. This process is summarized by the following steps:

1. Using Equations (6), (8) and (20), we calculate the shape and focal plane intensity distribution of a star, given its stellar magnitude. This gives the ideal intensity value of each pixel within the star image, as would be detected by the image detector.
2. The ideal signal from Step 1 is then combined with two typical imaging noise sources: shot noise and detector read noise. Shot noise describes a random variation in the observed amount of photoelectrons, due to the discrete, quantum nature of light. It is typically modeled as a Poisson distribution with *λ*(*x*,*y*) = *S_b_*(*x*,*y*). Read noise is essentially a summation of typical image detector noise sources and is generally modeled as a zero-mean, normally distributed random variable with *σ* = 3.5*e*^−^, where *e*^−^ denotes electrons.
3. The resulting image is quantized in two steps. First, a scaling parameter of 6.5 detector counts/*e*^−^ is applied. This corresponds to the gain of the ST-16's image detector. Second, the signal is converted into a 12-bit integer to reflect the 12-bit ADCs of the ST-16's image detector.
4. The last part of the process applies the ST-16 detection routine, described in Section 1.2, to determine if the star would be detected, and if so, measure its integrated intensity.

**Figure 8.**
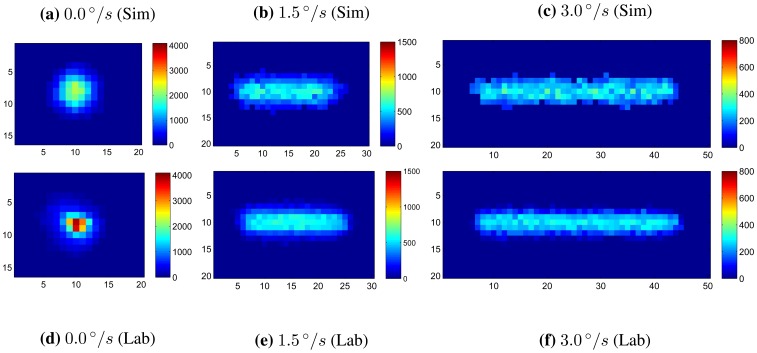
Simulated and lab-based images of a *m* ≈ 3.5 star at varying slew rates.

**Figure 9.**
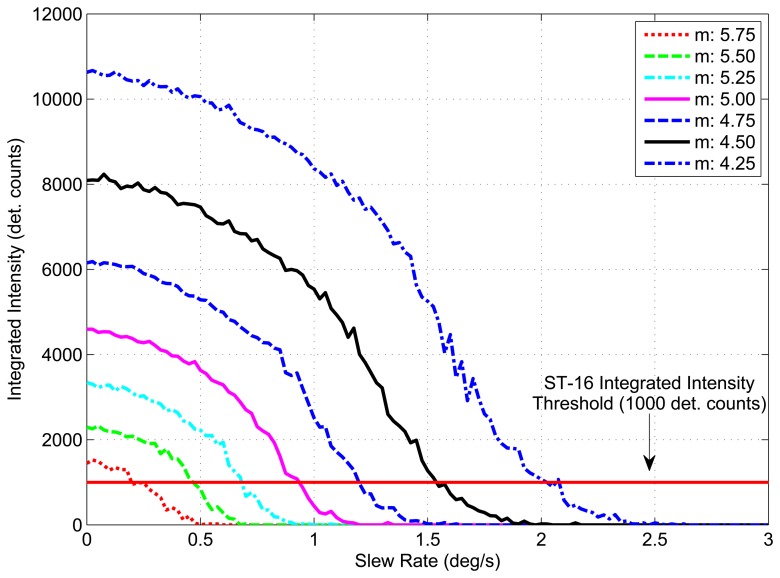
Simulated star intensity at varying slew rates (Reed sensitivity model).

Figure 8 shows examples of simulated star images for a *m* ≈ 3.5 star at slew rates of 0 °/*s*, 1.5 °/*s* and 3.0°/*s*. These are compared with lab-based star images taken using an ST-16 engineering model, a star source and a motorized gimbal (see Section 3.2 for details). Utilizing the process summarized above, images were simulated for stars of varying stellar magnitudes at slew rates of 0°/s to 10°/s. At each slew rate, the integrated intensity was recorded as measured by the ST-16 detection routine. Figure 9 shows the measured integrated intensity as a function of increasing slew rate for a set of stellar magnitudes, where *m* defines the intensity of each star as per Equation (8). The line at the bottom of the graph represents the integrated intensity threshold of the ST-16. Using the Liebe sensitivity model, given by Equation (6), instead of the Reed model, only changes the corresponding stellar magnitude labels. The trend in integrated intensity as a function of slew rate remains identical.

The trend of decreasing integrated intensity with increasing slew rate is similar for each stellar magnitude. The rate of this decrease in integrated intensity, denoted as the *loss rate*, is shallow at the beginning and gradually increases with increasing slew rate. At a particular slew rate, which varies depending on the brightness of the star, the loss rate reaches a maximum value, after which (for larger slew rates) it begins to decrease. This overall trend in changing loss rates of integrated intensity is due to the shape of the star, which is modeled as a symmetric Gaussian elongated along the direction of the slew rate. Since most of a star's intensity is concentrated at the centroid, the loss of integrated intensity for increasing slew rates is gradual for small slew rates. However, once the peak of the star smear begins to reach the lit pixel threshold, a large amount of lit pixels can be suddenly lost. The only remaining lit pixels are those closest to the centroid track, which typically contain substantially more intensity than their immediate neighbors. The intensity within these center pixels, can initially be hidden, due to the effects of pixel saturation. As the light from a saturated pixel is spread across a region of several pixels, an instantaneous increase can sometimes be seen in the integrated intensity of a star, as previously undetected light is now detected by the neighboring pixels; see Figure 9.

**Figure 10.**
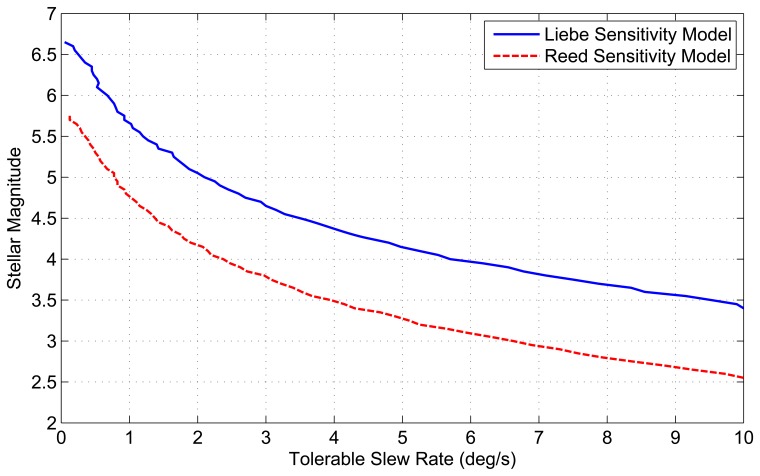
Tolerable slew rates for ST-16 star detection.

**Figure 11.**
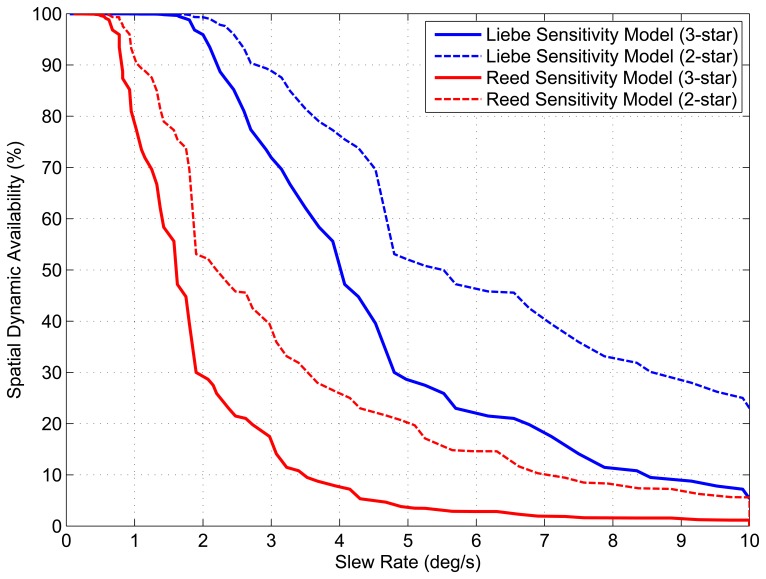
ST-16 spatial dynamic availability.

The results of these simulations are used to determine the maximum tolerable slew rate for a given stellar magnitude. This is achieved by examining when the ST-16 detection algorithm loses a star of a given magnitude. Figure 10 shows the tolerable slew rate for various stellar magnitudes (using both sensitivity models), up to a maximum slew of 10 °/s. Using the tolerable slew rate, we also calculate the spatial dynamic availability for both the *N_min_* = 3 (LIS) and *N_min_* = 2 (tracking) attitude solutions as shown in Figure 11. This calculation is just a repetition of the same method described in Section 1.2 for spatial static availability, except now with a stellar detection threshold based on Figure 10.

Despite the gradual trend in the tolerable slew rates for a given stellar magnitude, given by Figure 10, the corresponding drops in spatial availability are much more severe. We see that at a slew rate of 1 °/s, the spatial dynamic availability of the ST-16, as determined using the Reed-based sensitivity model, is ≈ 80% for a three-star (LIS) solution and ≈ 90% for a two-star (tracking) solution. Following this, we see the spatial dynamic availability fall to ≈ 30% for a three-star and ≈ 55% for a two-star solution, with an increase in the slew rate to 2 °/*s*. The corresponding trends for the Liebe-based results are better, with ≈ 100% availability at 1 °/*s* and > 90% availability at 2 °/*s* for both LIS and tracking solutions. However, the Liebe-based results also show a significant (but less abrupt) drop in dynamic spatial availability as the slew rate increases from 2 °/*s*–5 °/*s*.

### 3.2. Lab Tests

To evaluate the accuracy of the simulation tests, we have also conducted a series of lab trials using n ST-16 engineering model, a three-axis motorized gimbal and a star source. The motorized gimbal is constructed from Newport high-performance precision rotation stages (RSV240PP and RSV120PP) and a Newport C8 Motion controller. The repeatability of the rotation stages is 0.001°. The star source is a fiber-coupled tungsten halogen lamp, manufactured by Ocean Optics, and has a color temperature of a black body radiator at 2,800 K. To simulate stars of different stellar magnitudes, we adjusted the intensity of the star source with the use of neutral density filters and a variable attenuator. The lamp illuminates a 25 *μ* m pinhole, which is collimated by a telescope. When imaged by the ST-16, this star source has an apparent diameter of 12 pixels on the image detector (2.64 × 10^−5^ m).

Each trial consisted of testing the detection performance of a given stellar magnitude at a range of slew rates. The intensity model given by Equation (8) was used to match the static response of the ST-16 to the desired stellar magnitude. For each intensity, 30 images were taken at each slew rate spanning from 0 °/s to 3 °/s in 0.25 °/s steps, see Figure 8 for some example lab-based star images at rate. The mean value of these 30 measures of integrated intensity was then used for each angular rate to mitigate the random effects of shot noise and read noise.

**Figure 12.**
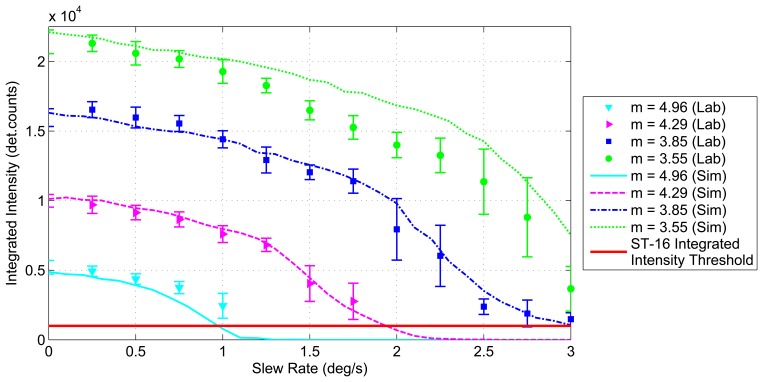
Lab measured star intensity at varying slew rates (Reed sensitivity model). Sim, simulation.

The results of these tests are displayed in Figure 12. Markers indicate the integrated intensity measured during lab trials, denoted in the figure as *Lab*. Error bars corresponding to each trial show the 1-sigma variation from the mean. Full lines represent results attained through simulation for corresponding beginning star brightnesses. These simulation-based results are denoted in the figure as *Sim*. There is strong correspondence between the intensity levels measured through simulation and lab trials.

### 3.3. Field Tests

The last method we examine for assessing detection performance is the use of field images. For static tests, field trials generally involve taking a star tracker out on a clear night and imaging the stars under the night sky. For assessing the tolerable slew rate, additional equipment (such as a motorized tripod mount) is required to move the star tracker at a precise angular rate while imaging. Several problems exist with both types of testing that can cause significant discrepancies in the measured integrated intensity of imaged stars. In this section, we examine these inaccuracies and how they impair our ability to get useful measurements of detection performance.

Several environmental factors associated with static field trials can impair our measurements of the integrated intensity of a given star. These include, but are not limited to: scintillation, high altitude cloud cover, aerosols and light pollution. Careful selection of a testing site far from any bright lights (cities) can effectively minimize the effect of the last of these error sources. However, scintillation, high altitude cloud cover and aerosols cause effects that continuously vary with time and, therefore, are harder to remove. Scintillation causes rapid variations in the apparent brightness of a celestial body, due to turbulence in the Earth's atmosphere. This can cause a star to appear brighter or dimmer than it nominally would. Due to the fact that this effect is a result of Earth's atmosphere, it cannot be avoided with field trials. However, the mean value of multiple intensity measurements of the same star over a short period of time can be used to increase the accuracy of the intensity measurement. Cloud cover and aerosols have a continuously varying attenuation effect on the measured integrated intensity of a given star. Atmospheric extinction modeling can help mitigate the effects of atmospheric attenuation on the measured intensity of detected bright stars [20–22]. However, atmospheric attenuation will degrade detection performance, preventing dim stars from being detected at all. Since the tolerable slew rate is based on finding the dimmest detectable star at a given rate, any degradation of detection performance directly impacts the determined tolerable slew rate. Additionally, atmospheric extinction models are highly variable and depend on several meteorological factors that can be difficult to measure. In many cases, a combination of careful planning, monitoring of forecast weather conditions and atmospheric modeling can help mitigate the effects of atmospheric attenuation, but they cannot be removed completely.

**Figure 13.**
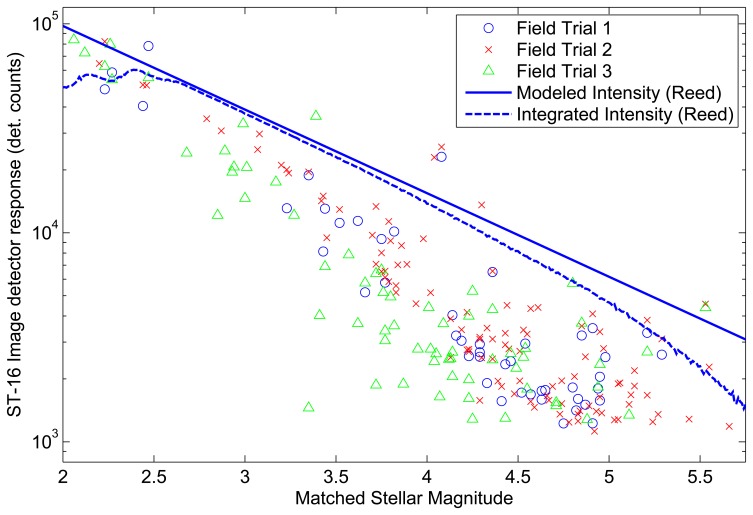
A comparison of star intensity measurements from different field observations.

In addition to environmental factors, several internal factors can contribute to the inconsistency in measured integrated intensity. Most notable are the effects of optical aberrations. These effects lead to changes in the size and shape of the imaged star as a function of off-axis distance. As discussed earlier in the paper, changes in the size and shape of the PSF directly impact the measured integrated intensity of the star. Given that any useful field image contains many stars that are generally located at several different off-axis angles, this effect introduces variations, even within a single image. As an example of the types of described variations, Figure 13 shows the results of three different field trials compared to the intensity model given by Equation (8). Field Trials 1 and 2 were taken a single day apart in two different locations, both of which were located a great distance away (>50 km) from any surrounding bright lights and were taken on days for which the cloud cover was reported to be clear. Field Trial 3 was taken several months later at a location approximately 20 km from a major city center and was also taken on a day for which the cloud cover was reported to be clear. In each case, the star tracker was pointed within 10 ° of zenith.

Figure 13 shows the variability present in measurements of integrated intensity from field trials, even in static conditions. Under dynamic conditions, the errors introduced by these variations quickly overcome the effects of the slew rate. This severely impairs the accuracy of measuring the tolerable slew rate for any given stellar magnitude. This method can still be used as a course validation of detection performance, but the result will be a conservative estimate of the actual integrated intensity of a star.

## 4. Along-Track Dynamic Availability

**Figure 14.**
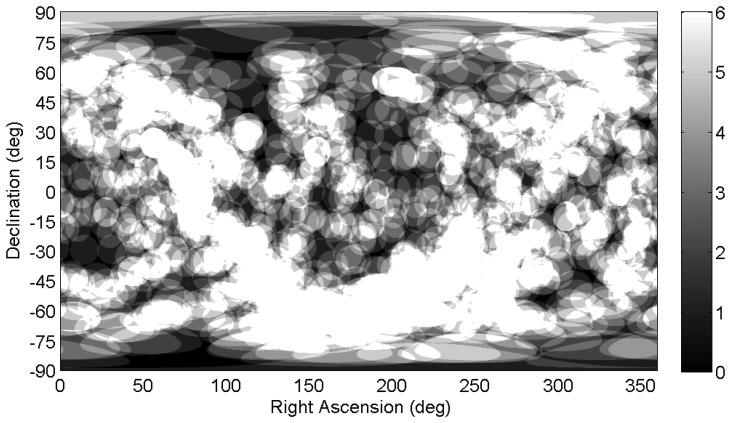
Spatial dynamic availability map of the ST-16 star tracker (1 °/s). Note: the color axis is flipped with respect to Figure 4 to increase the visibility of star-sparse regions.

Up until now, we have been discussing star tracker availability based on a spatial assessment of detectable stars over the entire celestial sphere. Although this measure of availability is useful for generalizing performance, in an actual mission, a star tracker will only be viewing a small portion of the celestial sphere, which is determined by the mission parameters of the host spacecraft. Since the distribution of stars along the celestial sphere is not uniform, the availability of the star tracker within this subset of the celestial sphere can differ substantially from the calculated spatial availability. This discrepancy is increased if we then include the effects of slew rate. To illustrate this, Figure 14 shows the number of detectable stars within the ST-16 FOV as a function of orientation while slewing at 1 °/*s*. If we compare Figure 14 with Figure 5, we can see that at a slew rate of 1 °/*s* there are significantly more views where the ST-16 will detect less than three stars. Figure 11 shows the calculated spatial dynamic availability of the ST-16 slewing at 1 °/*s* as approximately 80%. However, it is not difficult to imagine various types of missions where the orientation track of the star tracker across the celestial sphere would either: (a) include many star-sparse regions and, therefore, be less than 80%; or (b) exclude these star-sparse regions and have an availability greater than 80%. In this section, we examine the variation of star tracker availability along several simple orientation tracks and compare these results with the calculated spatial dynamic availability.

**Figure 15.**
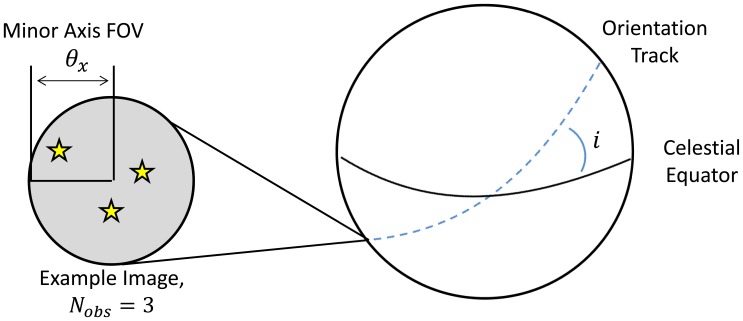
Schematic of orientation tracks for along-track dynamic availability.

Each orientation track is defined by an inclination, *i*, with respect to the celestial equator and represents a great circle on the celestial sphere; see Figure 15. We sample the orientation track at *N*_track_ = 1, 000 equally spaced orientations. At each orientation, we utilize the tolerable slew rates defined by the Reed-based sensitivity model *(N_min_* = 3) in Figure 10 to determine the number of stars that would be detected within the star tracker image, *N_obs_*, and if this satisfies the required number of stars for the star tracker attitude solution, *N_min_*.

Repeating this calculation for all sample orientations gives the fraction of the orientation track over which an attitude solution is possible. We denote this fraction as the along-track dynamic availability of the given orientation track. We repeat this analysis for different orientation tracks defined by (−90° ≤ *i* ≤ 90°), at constant slew rates |***ω***| = 1.0°/*s*, 1.5°/*s*, 2.0°/*s* and 3.0°/*s*.

**Figure 16.**
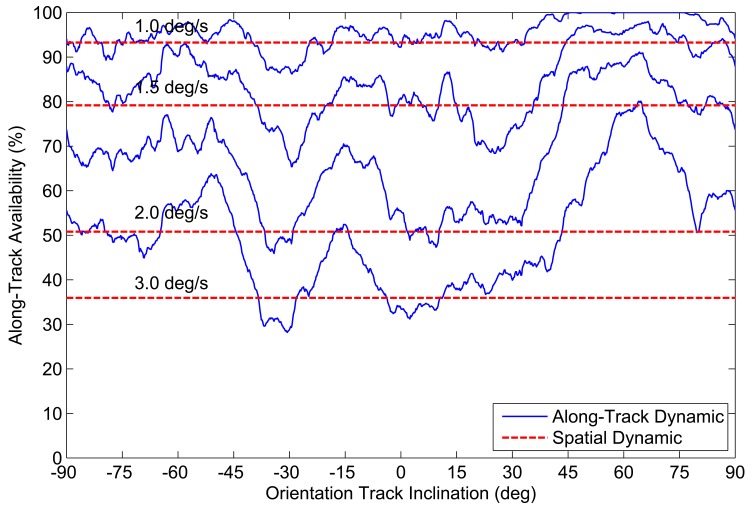
Examples of ST-16 along-track dynamic availability, *N_min_* = 2.

**Figure 17.**
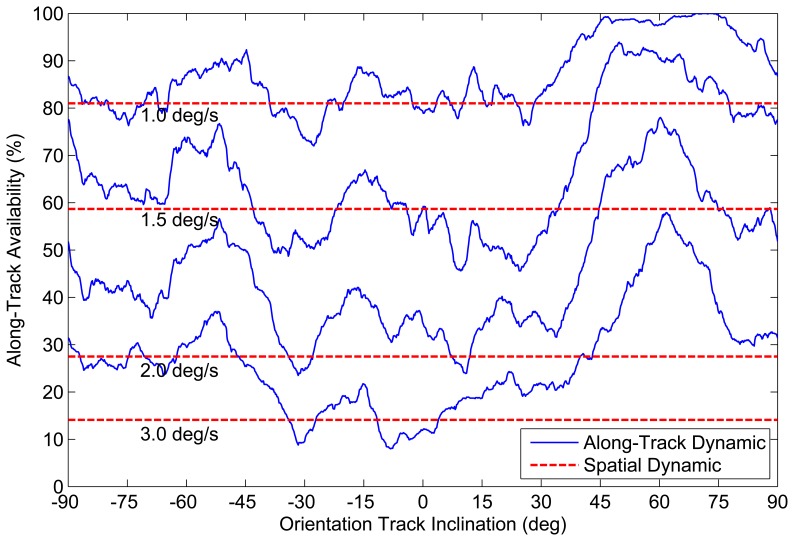
Examples of ST-16 along-track dynamic availability, *N_min_* = 3.

Figures 16 and 17 and Table 2 show the variation in along-track dynamic availability as a function of slew rate and track inclination for both tracking (*N_min_* = 2) and LIS (*N_min_* = 3) attitude solutions. For comparison, we overlay a series of horizontal lines that indicate the determined spatial dynamic availability (from Figure 11) at each respective slew rate. We can see from Figures 16 and 17 that along-track dynamic availability varies quite significantly as a function of the specific path chosen. Just from the example missions shown, we see that the along-track dynamic availability can differ from the calculated spatial dynamic availability by more than 15%.

Determining the along-track dynamic availability of a star tracker can be a challenging task. One needs to model the mission dynamics quite accurately to be able to predict the path the sensor will follow along the celestial sphere. In addition, one needs to establish the relationship between the stellar magnitude of a given star and the tolerable slew rate (see Figure 10). Given that the latter task is independent of the selected mission and has been shown to be measurable using lab/ground tests, it could be provided by the sensor manufacturer. If these tasks can be achieved, even with a simplified set of dynamics, some rough bounds on star tracker availability can be determined. These can then be used as a coarse tool for star tracker feasibility studies and/or trade studies for star tracker placement.

## 5. Conclusions

The main goal of this paper was to increase the fidelity of star tracker availability modeling by including the effects of slew rate and star tracker detection logic. We have achieved this by a three-part solution. First, we formulated an analytical model to describe the effects of slew rate on the focal plane intensity distribution of a star. Second, we used this model to relate slew rate to star tracker detection performance, through simulations, which were verified by lab tests. Third, we used this determined relationship between detection performance and slew rate to calculate star tracker availability under dynamic conditions.

Good correspondence was seen between the results from the simulations and those from lab tests; see Figure 12. Even the conservative sensitivity model indicated that the ST-16 satisfies the design requirement of maintaining high availability (>80%) while tracking a ground target from low Earth orbit (LEO) (slew rate ≈ 1 °/*s*). In comparison, the Liebe-based sensitivity model indicated that we can maintain >90% availability at slew rates of up to 2 °/*s*. Field trials were shown to be a poor choice for measuring the tolerable slew rate. This is due to variations in the measurable integrated star intensity caused by several parameters internal and external to the sensor.

We finished the paper with a brief examination of along-track dynamic availability for a set of simple mission dynamics. Although along-track results do vary from the calculated spatial dynamic availability, the latter can serve as a conservative first-cut approximation of star tracker availability performance at a rate. The calculation of along-track dynamic availability requires knowledge of mission details and the relationship between the tolerable slew rate and stellar magnitude. Using methods described in this paper, we can achieve the latter part of this solution. If one can then attain even a simplified understanding of the expected mission dynamics, we can begin to form bounds on the availability performance of a star tracker.

## Figures and Tables

**Table 1.** Key ST-16 Specifications.

Accuracy	0.01° with 85% confidence
Size	59 × 56 × 31.5 mm
Mass	≈ 90 g
Field of View	7.5° half-axis
Exposure Time	100 ms
Detection Threshold	<5.75
Catalog	3,746 stars
Availability (Static)	>99.99%
Lens Diameter	D = 12 mm
Lens F#	1.2

**Table 2.** ST-16 Along-track Dynamic Availability Statistics (*N_min_* = 2 / *N_min_* = 3).

**Slew Rate (°/s)**	**Spatial Dynamic Availability (%)**	**Along-Track Dynamic Availability (%)**		
		**Mean**	**Max**	**Min**
1.00	93.3 / 80.9	95.5 / 86.9	100.00 / 100.0	86.2 / 72.0
1.50	79.1 / 58.6	84.5 / 66.6	98.0 / 93.9	65.4 / 45.6
2.00	50.8 / 27.6	67.7 / 45.3	91.1 / 78.0	45.9 / 23.5
3.00	38.9 / 14.2	49.8 / 26.6	80.1 / 57.9	28.2 / 8.0
